# Preoperative Dental Examination Might Prevent Unnecessary Tooth Extractions During Dental Treatment of Patients with Disabilities Under General Anaesthesia – Results of a Retrospective Cross-Sectional Study

**DOI:** 10.3290/j.ohpd.a43364

**Published:** 2020-02-12

**Authors:** Gerhard Schmalz, Mathias Farack, Tanja Kottmann, Jana Schmidt, Felix Krause, Dirk Ziebolz

**Affiliations:** a Dental Assistant, Department of Cariology, Endodontology and Periodontology, University of Leipzig, Germany. Wrote the manuscript and contributed substantially to analysis and interpretation of data.; b Dentist, Department of Cariology, Endodontology and Periodontology, University of Leipzig, Germany. Organised and performed data management; co-wrote and reviewed the manuscript.; c Statistician, Kottmann & Co., Hamm, Germany. Performed the overall data analysis and statistics and reviewed manuscript.; d Dental Assistant, Department of Cariology, Endodontology and Periodontology, University of Leipzig, Germany. Interpreted the data and critically revised the manuscript for important intellectual content.; e Assistant Professor, Department of Cariology, Endodontology and Periodontology, University of Leipzig, Germany. Co-wrote and reviewed the manuscript and gave final approval.; f Assistant Professor, Department of Cariology, Endodontology and Periodontology, University of Leipzig, Germany. Head of the study, designed research and data analysis, interpreted the data and reviewed manuscript.; * These authors contributed equally as first author.

**Keywords:** disability, dental treatment, dental examination, general anaesthesia, dental care

## Abstract

**Purpose:**

The aim of this retrospective cross-sectional study was to detect dental health and dental treatment under general anaesthesia, as well as associations to selected parameters in a patient cohort with different disabilities.

**Material and Methods:**

Patients with disabilities, including mental, physical, combination of mental and physical as well as psychiatric disability, which received dental rehabilitation under general anaesthesia between 1 January 2002 and 31 December 2011 were included. Based on the available patients’ records, findings of dental examination (Decayed-, Missing- and Filled-teeth index [DMF-T]), treatment documentation as well as further specific factors including the presence of preoperative dental examination or radiographs were analysed. Statistical analysis was conducted using Mann-Whitney U test, Kruskal–Wallis test, analysis of variance (ANOVA), chi-squared or Fisher’s exact test (p <0.05).

**Results:**

A total of 464 patients were included. An overall DMF-T of 12.3 ± 7.5 (D-T of 5.8 ± 5.1) and a dmf-t of 9.2 ± 5.0 (d-t of 7.5 ± 4.5) were found. Patients with psychiatric disabilities showed worst dental health. About half of patients (56%) received a professional tooth cleaning. A tooth extraction was executed at 70% of patients, with 3.3 ± 4.5 teeth each patient. Nearly no patient received periodontal or endodontic treatment. Patients with a preoperative dental examination received statistically significantly less tooth extractions compared to patients without preoperative dental examination (2.7 ± 3.7 vs 4.5 ± 5.8).

**Conclusion:**

Patients with different disabilities show high dental treatment need and require improved dental care. Thereby, the preoperative dental examination might avoid unnecessary tooth extractions and is therefore strictly recommended.

According to the World Health Organization (WHO) report on disabilities in 2011, about 15% of the world population shows any form of disability, whereby 110–190 million individuals were reported to have a severe condition.^22^ These patients are often limited in their daily life, which is also potentially related to oral and dental health issues. Accordingly, patients with disabilities are often affected by the presence of cognitive, physical and behavioural limitations that compromise individuals in their daily oral care and cooperation during dental visits.^7,8,13,17^ Furthermore, the patients often take several medications that may influence oral health.^5,7-9,13,17^ Moreover, elevated rates of poverty have been reported.^6^

These special characteristics lead to two major concerns in this patient group. On the one hand, these patients often show an insufficient oral status, including a high prevalence of dental and periodontal diseases.^4,14,16,20^ On the other hand, dental treatment is preponderantly difficult; the patients are often orally defensive or show acute situational anxiety reactions during dental treatment. Therefore, the ability of the patients to comply with treatment is limited.^1,18^ Accordingly, a sufficient dental treatment is often restricted to a therapy under general anaesthesia, with its related costs and burden for the patients and their caregivers.^1,18^

Taking these special characteristics into account, the repeatedly formulated demand of improvements in dental care for this patient group is comprehensible.^2,12,14^ However, the real situation of established measures in dental care is still not completely clarified, and potential adjusting screws are still unclear. Especially, the regular dental rehabilitation of patients with disabilities during general anaesthesia needs further evaluation to detect both, the present dental treatment need and resulting therapy. Accordingly, the current study should detect these parameters to identify potential opportunities to improve the dental care under general anaesthesia in this patient group.

Therefore, the aim of this retrospective investigation was to detect the dental health of a patient cohort with different disabilities undergoing dental treatment under general anaesthesia. Furthermore, the dental treatment performed under general anaesthesia, as well as potential associations to different selected parameters, should be examined. It was hypothesised that the overall treatment need would be high, and the therapy would be associated with the presence of preoperative dental examination and the form of disability.

## Material and Methods

### Study Design

This study was designed as a retrospective, monocentric, cross-sectional study. The study was reviewed and approved by an ethics committee (application no. 2/6/12). The patients, as well as the authorised person, were informed verbally and in writing about scientific use of the clinical and treatment data (independent of this study), and gave their written informed consent.

### Patients

Patients with different disabilities, including: (A) mental disability; (B) physical disability; (C) combination of mental and physical disability; and (D) severe psychiatric disease/psychiatric disability were included in the study. Thereby, patients treated under general anaesthesia between 1 January 2002 and 31 December 2011 in a university dental clinic were selected by previously defined inclusion and exclusion criteria. Mandatory condition for inclusion was the affiliation to one of the four groups (A–D) and treatment within the study period. Children, adolescents and adults were considered. The only exclusion criterion was the incompleteness of the treatment documentation.

### Procedure of Dental Rehabilitation Under General Anaesthesia

All patients were dentally rehabilitated in the university dental clinic by different experienced dentists during the investigation period. Every patient attended the responsible dentist for treatment at a preoperative appointment, whereby a dental examination and/or radiographs were performed as far as possible. Afterwards, patients were allocated to an anaesthesiologist and an appointment for the rehabilitation under general anaesthesia was arranged. During the dental rehabilitation, patients received dental treatment according to their treatment need. Every treatment was documented in the patient’s records.

### Data Acquisition

Patients meeting the in- and exclusion criteria were selected from the patients’ documentation of the department. Based on the available patients’ records, the findings of the dental examination, the treatment documentation as well as further specific factors including age, gender, waiting time until rehabilitation, duration of operation, presence of preoperative dental examination or radiographs were recorded. As results for dental examination, the Decayed-, Missing- and Filled teeth index (DMF-T) or Decayed-, Missing- and Filled surface index (DMF-S) was assessed. This index reflects the presence of teeth/surfaces showing a carious cavitation of the tooth surface (D-component), the presence of missing (M-component) and filled (F-component) teeth. For children of mixed denture, the adapted dmf-t/dmf-s was applied.^21^ Based on the available findings, patients were allocated into one of the subgroups (A–D).

### Statistical Analysis

All statistical analyses were performed with SPSS Version 24.0 (SPSS, IBM, New York, USA). The metrical variables were tested for their normal distribution with Kolmogorov–Smirnov test, whereby dmf-s and dmf-t were found to be normal distributed (p >0.05), while all other parameters did not show normal distribution (p <0.05). For comparison of two parameters, Mann–Whitney U test was used. For comparing more than two independent, non-normal distributed samples, Kruskal–Wallis test was applied. ANOVA was used for more than two independent, normal distributed variables. Categorical data were analysed with either chi-squared or Fisher’s exact test. The level of statistical significance was set at p <0.05.

## Results

### Patients

A total of 464 patients could be included in the study: group A = 145, group B = 53, group C = 216, group D = 50. The patients in group D (severe psychiatric disease/psychiatric disability) were significantly older than groups A–C (p <0.01). All patients’ characteristics including waiting time and duration of operation are given in Table 1.

**Table 1:** Patient characteristics

Parameter	Total (n = 464)	Group A (n = 145)	Group B (n = 53)	Group C (n = 216)	Group D (n = 50)
**Age in years** (mv ± sd)	23.5 (28)	27.0 (25)	14.0 (28)	18.0 (22)	62.0 (37)
**Body-Mass-Index** (BMI: kg/(m²) (mv ± sd)	20.95 (9.7)	21.2 (10.4)	19.2 (9.0)	20.9 (8.6)	22 (9.2)
**Duration of operation in min** (mv ± sd)	115 (79)	122 (78)	119 (122)	112 (71)	130 (105)
**Waiting time until operation in days** (mv ± sd)	100.5 (87)	110 (90)	116 (96)	96.5 (88)	65.5 (94)

### Oral Status

In adult participants, an overall DMF-T of 12.3 ± 7.5 was detected, whereby a D-T of 5.8 ± 5.1 and an M-T of 2.9 ± 4.0 was found. Between the subgroups, group D was found to show a higher DMF-S, D-S, M-S, DMF-T, D-T and M-T compared to the other subgroups A–C (p <0.01, Table 2). In comparison of the other subgroups (A–C), no statistically significant differences in dental findings were detected (p >0.05). For children and adolescents in mixed denture, a dmf-t of 9.2 ± 5.0, a d-t of 7.5 ± 4.5 and an m-t of 1.4 ± 2.4 was detected, without statistically significant differences between subgroups A–C (p >0.05, Table 3). Because no children were part of group D, no dmf-t values are presented for this subgroup.

**Table 2:** Dental findings of adult participants including decayed-missing-filled-surface (DMF-S) and decayed-, missing-, filled-teeth (DMF-T) index

		Total (n = 464)	Group A (n = 145)	Group B (n = 53)	Group C (n = 216)	Group D (n = 50)	p value
**DMF-S** (mv ± sd)	**n =**	253	90	22	121	20	–
	**DMF-S**	33.8 ± 27.9	35.8 ± 25.1	32.0 ± 27.7	27.0 ± 24.3	67.9 ± 35.7	<0.01
	**D-S**	13.5 ± 16.0	12.5 ± 15.1	20.73 ± 16.3	9.8 ± 13.3	32.2 ± 20.6	<0.01
	**M-S**	13.1 ± 18.5	15.5 ± 16.5	9.1 ± 15.3	9.9 ± 16.1	25.7 ± 33.1	<0.01
	**F-S**	7.7 ± 10.2	8.0 ± 10.2	3.0 ± 4.5	7.7 ± 9.4	11.8 ± 16.9	0.12
**DMF-T** (mv ± sd)	**n =**	241	88	23	109	21	–
	**DMF-T**	12.3 ± 7.5	13.2 ± 7.0	12.0 ± 6.4	10.4 ± 7.3	19.3 ± 7.14	<0.01
	**D-T**	5.8 ± 5.1	5.7 ± 5.2	8.2 ± 4.9	4.5 ± 4.3	9.9 ± 6.0	<0.01
	**M-T**	2.9 ± 4.0	3.3 ± 3.4	2.0 ± 3.2	2.2 ± 3.7	5.5 ± 6.9	<0.01
	**F-T**	3.7 ± 4.6	4.3 ± 5.2	1.7 ± 2.3	3.9 ± 4.4	3.7 ± 4.2	0.25

mv: mean value, sd: standard deviation.

**Table 3:** Dental findings of children and adolescent participants including decayed-, missing-, filled-surface (dmf-s) and decayed-, missing-, filled-teeth (dmf-t) index

		Total (n = 464)	Group A (n = 145)	Group B (n = 53)	Group C (n = 216)	Group D (n = 50)	p value
**dmf-s** (mv ± sd)	**n =**	79	18	18	43	–	
	**dmf-s**	26.0 ± 16.2	25.1 ± 17.9	29.2 ± 13.5	24.7 ± 16.6	–	0.25
	**d-s**	20.1 ± 14.9	19.3 ± 12.8	22.8 ± 16.0	19.3 ± 15.4	–	0.60
	**m-s**	5.5 ± 10.1	5.7 ± 10.9	6.1 ± 9.2	5.1 ± 10.3	–	0.77
	**f-s**	0.9 ± 4.6	0.4 ± 1.2	0.4 ± 1.0	1.3 ± 6.2	–	0.97
**dmf-t** (mv ± sd)	**n=**	80	17	17	46	–	
	**dmf-t**	9.2 ± 5.0	7.9 ± 4.3	10.2 ± 3.4	9.3 ± 5.7	–	0.43
	**d-t**	7.5 ± 4.5	6.4 ± 3.2	8.3 ± 3.8	7.5 ± 5.1	–	0.46
	**m-t**	1.4 ± 2.4	1.4 ± 2.6	1.7 ± 2.2	1.3 ± 2.5	–	0.66
	**f-t**	0.3 ± 1.2	0.1 ± 0.3	0.3 ± 0.5	0.5 ± 1.6	–	0.97

mv, mean value, sd, standard deviation.

### Dental Treatment

For 70% of participants, a preoperative dental examination was available, 67% had at least one dental radiograph. About half of patients (56%) received a professional tooth cleaning, whereby group C had the highest amount of professional tooth cleaning (p <0.01). With 1.8 ± 3.2, group D received the least plastic restorations between subgroups (p <0.01). A tooth extraction was executed at 70% of patients, with 3.3 ± 4.5 teeth each patient. With 88% of patients with on average 7.4 ± 7.2 teeth, group D showed most tooth extractions (p <0.01). Nearly no patient received periodontal or endodontic treatment. Furthermore, 25% of all patients received a further treatment under general anaesthesia during the investigated time period (Table 4).

**Table 4:** Different treatment measures which were performed during general anaesthesia

			Total (n = 461)	Group A (n = 145)	Group B (n = 53)	Group C (n = 213)	Group D (n = 50)	p value
**Preoperative dental examination** (%[n])			70% (323)	72% (105)	77% (41)	72% (156)	42% (21)	<0.01
**Radiographs** (%[n])			67% (309)	67% (97)	57% (30)	64% (138)	88% (44)	<0.01
**Plastic restorations** (mv ± sd)	**total**		3.4 ± 4.3	3.4 ± 3.8	4.7 ± 5.5	3.5 ± 4.5	1.8 ± 3.2	<0.01
	**F1**		2.0 ± 3.0	1.9 ± 2.5	2.6 ± 3.7	2.2 ± 3.2	0.7 ± 1.8	<0.01
	**F2**		0.8 ± 1.4	0.9 ± 1.5	1.00 ± 1.5	0.8 ± 1.4	0.6 ± 1.2	0.33
	**F3**		0.4 ± 1.0	0.3 ± 0.8	0.7 ± 1.3	0.4 ± 1.0	0.3 ± 0.8	0.10
	**F4**		0.3 ± 1.0	0.3 ± 0.9	0.5 ± 1.5	0.2 ± 0.9	0.10 ± 0.37	0.95
**Professional tooth cleaning** (%[n])			56% (260)	61% (89)	19% (10)	62% (131)	28% (14)	<0.01
**Periodontal therapy** (%[n])			2% (9)	1% (2)	0% (0)	3% (7)	0% (0)	0.24
**Tooth extractions** **Each patient** (mv ± sd)		**Patients** (%[n])	70% (326)	64% (93)	79% (42)	68% (147)	88% (44)	0.01
		**each patient** (mv ± sd)	3.3 ± 4.5	2.8 ± 4.1	3.4 ± 3.4	2.6 ± 3.7	7.4 ± 7.2	<0.01
**Endodontic treatment** (%[n])			2% (11)	3% (4)	2% (1)	3% (6)	0 (0)	0.67
**Further treatment under general anaesthesia during study period** (%[n])			25% (116)	32% (46)	11% (6)	28% (61)	6% (3)	<0.01

mv, mean value, sd, standard deviation.

Overall, patients with a preoperative dental examination received statistically significantly less tooth extractions compared to patients without preoperative dental examination (2.7 ± 3.7 vs 4.5 ± 5.8, p <0.01). The presence of dental radiographs was not associated to the number of extractions (3.6 ± 4.9 vs 2.7 ± 3.5, p = 0.16).

## Discussion

### Summary of the Main Results

Investigated patients with disabilities showed a largely insufficient dental status, whereby patients with severe psychiatric disease/psychiatric disability were found to present the highest prevalence of caries. During treatment, patients received plastic restorations and tooth extractions, while only half of patients received a professional tooth cleaning. Furthermore, nearly no patient had an endodontic or periodontal treatment. The presence of a preoperative dental examination was statistically significantly associated to the number of extracted teeth.

### Comparison of the Findings with the Literature

The recent literature suggests both a high burden of oral diseases as well as the necessity to improve dental care in patients with disabilities, regardless if they are children or adults.^2,14,16,19^ A comparable study, which also investigated German patients with disabilities, found a comparable DMF-T to the findings of the current study, but reasonably lower caries prevalence.^20^ The fifth German oral health study (DMS V), a representative study for German population found a DMF-T of 11.2 in adults of the General population, which is also comparable to the current study.^10^ However, the caries prevalence was considerably higher in the current study. For children, the DMS V found nearly no carious teeth and only a dmf-t of 0.5, which is a substantial difference to the current study in which children showed a high caries prevalence.^10^ These findings are completely in line with the literature.^2,14,16,19^ Therefore, the high dental treatment need in patients with disabilities appears evident. Moreover, the current study found higher caries prevalence in patients with psychiatric diseases. Differences in oral status between patients with different disabilities and resulting requirements between patients with different disabilities have already been reported.^12^ However, in the current study, the large age difference between psychiatric diseases patients and other subgroups can explain the differences in DMF-T. Accordingly, these subgroup differences might not attach any great importance to the effect.

Altogether, the dental treatment of these patients with a high dental treatment need is of relevance. Patients with disabilities often show a lack of accessibility to dental services.^11^ Thereby, an improvement in dental care with application of a multidisciplinary approach appears reasonable.^15^ However, the patients are often orally defensive or anxious, making a treatment under general anaesthesia necessary.^1,18^ It has been described that patients with disabilities receive extractions rather than restorative therapy.^3^ Furthermore, patients with disabilities were shown to receive more extractions and restorations than patients with systemic diseases in general anaesthesia rehabilitation.^18^ Accordingly, the current study aimed to detect the conducted dental treatment during general anaesthesia in the investigated patient group. It was found that plastic restorations and tooth extractions were the predominant therapeutic interventions, what is in line with the literature.^3,18^ In contrast endodontic and periodontal treatment was only executed in isolated case. Chang et al^1^ investigated the effect of dental rehabilitation under general anaesthesia on oral health-related quality of life and showed endodontic treatment to positively influence this parameter. Generally, endodontic and possibly periodontal therapy might allow tooth conservation, which could create a benefit in oral health-related quality of life. The absence of endodontic treatment in the current study could be due to the technical effort and time consumption required, but it could be a possible approach of improvement for future concepts. Furthermore, it is conspicuous that only half of the patients received professional tooth cleaning. Of course, this is potentially a sign for a lack of preventive strategy during dental rehabilitation of patients with disabilities under general anaesthesia and might explain that nearly one-quarter of the patients needed a further general anaesthesia treatment during the investigation period. However, this is just speculative and cannot be supported by the available data. But in principle, when a patient undergoes general anaesthesia anyway for dental rehabilitation, a preventive therapy including professional tooth cleaning is necessary, reasonable and should therefore be applied.

The most clinically relevant finding of the current study might be that patients with a preoperative dental examination received significantly less tooth extractions compared to patients without preoperative dental examination. Patients with disabilities often receive preferred and potentially premature tooth extractions instead of conservative therapy.^3^ This leads to increased prosthodontic treatment need, which mostly cannot be fulfilled due to the reduced compliance and treatability.^12^ Accordingly, in contrast to the regular procedure, a tooth extraction should be assessed critically. Of course, teeth with a potential risk for the general health should be extracted, but the possibility to conserve teeth (with restorative, endodontic and/or periodontal treatment) might help to increase patients chewing ability and quality of life. Considering the current study’s results, a preoperative examination helps to reduce extractions and therefore might prevent unnecessary extractions. This leads to the mandatory recommendation to execute a dental examination before dental treatment of patients with disabilities. If a preoperative examination is impossible, an intraoperative examination before treatment should be performed as a minimum. As the current study shows, preoperative radiographs are not helpful for reducing the extraction of teeth, but should also be applied where possible to assess patients’ endodontic situation.

### Strengths and Limitations

To the best of the authors’ knowledge, this is the first study highlighting the importance of a preoperative dental examination and giving clear recommendations for dental rehabilitation of patients with disabilities under general anaesthesia, focusing a conservative approach. The current study evaluates the dental health situation and received treatment of patients with disabilities under general anaesthesia. The inclusion of 464 participants with different disabilities is a clear strength of the study. Furthermore, it was possible to find an association between the presence of a preoperative dental examination and the number of tooth extractions. However, several limitations must be addressed. The design as a retrospective cohort study limits the ability to draw meaningful conclusions, especially the clinical indication of the performed therapy (eg, restoration or tooth extraction cannot be assessed retrospectively and must be considered in the interpretation of the findings). Moreover, the fact that qualified but different dentists conducted dental treatment during study period limits the current study. Furthermore, the large age difference between the groups strictly limits comparability between subgroups. A further point is the absence of a healthy control group. However, a healthy control would only be helpful for interpretation of the dental findings and not the dental treatment during rehabilitation, which was the main focus of the current study. For interpretation of dental findings, the DMS V as a representative study for German general population was used, making a healthy control dispensable.

## Conclusion

Patients with different disabilities show high dental treatment need and require improved dental care. In particular, as tooth extraction was found to be the major therapy, a conservative treatment should be prioritised. Thereby, a preoperative dental examination might avoid unnecessary tooth extractions and is therefore strictly recommended.
